# RETRACTION: The Protective Effects of Imperatorin on Acetaminophen Overdose-Induced Acute Liver Injury

**DOI:** 10.1155/omcl/9795689

**Published:** 2025-11-14

**Authors:** Oxidative Medicine and Cellular Longevity

RETRACTION: Z. Gao, J. Zhang, L. Wei, X. Yang, Y. Zhang, B. Cheng, Z. Yang, W. Gao, C. Song, W. Miao, K. Williams, C. Liu, Q. Xu, Y. Chang, Y. Gao, "The Protective Effects of Imperatorin on Acetaminophen Overdose-Induced Acute Liver Injury," *Oxidative Medicine and Cellular Longevity*, vol. 2020, 8026838, 2020, https://doi.org/10.1155/2020/8026838.

The above article, published online on 13 May 2020 in Wiley Online Library (wileyonlinelibrary.com), has been retracted by agreement between the journal Editor-in-Chief, Jeannette Vasquez-Vivar, and John Wiley & Sons Ltd. The retraction has been agreed due to errors in the 4th panel of Figure 3b, and the IMP + APAP, WT panels of Figures 5h and 6e. Specifically:

• The 4th panel of Figure 3b is duplicated with the 2nd panel of Figure 1f, with different exposure.
• The IMP (10 μM) + APAP, WT panel of Figure 6e overlaps with the ADAP (10 mM) panel of Figure 6e.
• The WT/ADAP (10 mM) panel of Figure 5h overlaps with the FXR-/- + APAP (10 mM) panel of Figure 5h.

The authors initially requested a correction for Figures 3 and 6. After requesting further clarification, they explained that during the experiment they captured several images with different microscopes and different people at various times to confirm the results of ROS fluorescent and TUNEL staining, but due to a mistake during the preparation of the manuscript, it was found that the KO group and normal group were incorrect.

The Chief Editor confirms that the errors render the results unreliable, and the manuscript is retracted.

During the author's initial correction request, the corresponding author requested for Kevin Williams to be removed as an author prior to the investigation due to lack of contribution to the study. Yongshen Chang requested to be removed as an author during the investigation.

Yong Gao disagrees with the retraction. Xu Qin, Kevin Williams and Li Wei agree to the retraction. No response was received from the remaining authors.

